# Inconsistency in the Crown-to-Root Ratios of Single-Rooted Premolars Measured by 2D and 3D Examinations

**DOI:** 10.1038/s41598-017-16612-x

**Published:** 2017-11-28

**Authors:** Hsiang-Hsi Hong, Heng-Liang Liu, Adrienne Hong, Pu Chao

**Affiliations:** 1 Physician attending, Department of Periodontics, Chang Gung Memorial Hospital and Chang Gung University, Linkou, Taiwan; 2 School of Dental Technology, College of Oral Medicine, Taipei Medical University, Taipei, Taiwan; 3 Research associate, Instrument Department, Chang Gung Memorial Hospital, Linkou, Taiwan; 4 Research associate, California Northstate University, College of Medicine, Elk Grove, CA United States; 5 Research associate, Case Western Reserve University, School of Dental Medicine, Cleveland Heights, OH United States

## Abstract

Micro-computed tomography (micro-CT) was applied to elucidate the relationship between the three-dimensional (3D) root surface area (RSA) and two-dimensional (2D) crown-to-root ratio (CRR) of extracted teeth to classify the periodontitis and assign a periodontal/prosthetic prognosis. A total of 31 maxillary and 35 mandibular single-rooted human premolars were examined. The amount of periodontal support on the basis of 3D RSA and 2D root length (RL) at CRRs of 1:1, 5:4, 3:2, and 2:1 were analyzed. Both maxillary and mandibular premolars demonstrated a nonsignificant RSA percentage at the evaluated CRRs. The coronal 21%–22% 2D RL and the 26%–28% 3D RSA bone loss apical to the cemento-enamel junction corresponded to a CRR of 1:1, relating to mild-moderate periodontitis. The coronal 30%–31% 2D RL and the 41%–42% 3D RSA bone loss corresponded to a CRR of 5:4, correlating to severe periodontitis. More severe clinical attachment loss (CAL) was observed in the 3D RSA measurement than in the 2D RL measurement at the evaluated CRRs. The amount of CAL at the CRR of 1:1 was inadequate to assess the severity of periodontitis on the basis of the 2D RL and 3D RSA measurements.

## Introduction

Dentists evaluate many clinical parameters, such as the amount of bone loss, probing depth, furcation involvement, tooth mobility, root form, pulpal involvement, therapist skill and knowledge, caries, tooth position and occlusal relationship, strategic valve, and crown-to-root ratio (CRR), to assign a prognosis to an individual tooth^1^. Clinicians employ two-dimensional (2D) linear CRR extensively as an objective index to examine whether the candidate’s abutment teeth are indicated for tooth-borne fixed and removable prostheses^2–5^. In the field of prosthetics, the CRR is defined as “the physical relationship between the portion of the tooth within the alveolar bone compared with the portion not within the alveolar bone, as determined radiographically”^6^. An abutment tooth with a higher CRR always involves a lower amount of supporting bone; therefore, it receives a higher loading force from occlusion and an unfavorable lateral force spread from removable partial dentures (RPDs)^7^. A consensus on the prognostic value of CRR in assigning a prosthetic prognosis is yet to be established^8,9^. Dykema^10^ proposed that a CRR of 1:2 was ideal and that a CRR of 2:3 was acceptable and desirable for fixed partial dentures (FPDs) on abutment teeth. In comparison, CRRs of 2:3 and 1:1 have been reported as the optimal and minimum ratios, respectively, for the abutment teeth on FPDs when the periodontium is healthy and occlusion is controlled^8,11,12^. Moreover, the quantitative evidence to support these values is limited.

The American Academy of Periodontology (AAP) guidelines define mild, moderate, and severe periodontitis as characterized by 15, 16–30%, and >30% radiographic bone loss^13^. Relying on the CRR value of 1:1, periodontists assign a favorable, cautious, or unfavorable prognosis to a tooth with periodontitis. However, the associations between the optimum CRR and the amount of radiographic bone loss have not been well documented. Furthermore, differentiating satisfactory from unsatisfactory CRR based on the 1:1 ratio to evaluate the diagnosis of mild, moderate, and severe periodontitis is discordant^1,14^.

Micro-computed tomography (micro-CT, SkyScan, Bruker, Kontich, Belgium) has been used to support the clinical findings of dental investigations^15–19^. After tooth scanning and software analysis, reconstructed three-dimensional (3D) files can be translated into 2D data for root surface area (RSA) analysis, and information on the RSA of the scanned tooth at the evaluated CRRs can be calculated. We hypothesized that the corresponding amounts of periodontal bone support at the evaluated CRRs based on the 3D RSA measurement are different from those based on the 2D root length (RL) measurement. The purpose of this study was to apply a micro-CT to scan extracted human single-rooted premolars and measure the 3D RSAs at the evaluated linear CRRs levels and to decide if the amount and ratio of the measured 3D RSAs were comparable to those assessed on the basis of 2D RL at same CRRs. The amount of clinical attachment loss (CAL), corresponding to 2D RL measurements, has been applied to diagnose the severity of periodontitis^13^. The 3D RSA and 2D RL ratios of CALs representing mild, moderate, and severe periodontitis were also analyzed. Finally, the prognostic values of CRR in periodontal and prosthetic appraisals in accordance with the 3D RSA and 2D RL ratios were surveyed and discussed.

## Results

**Figure 1:**
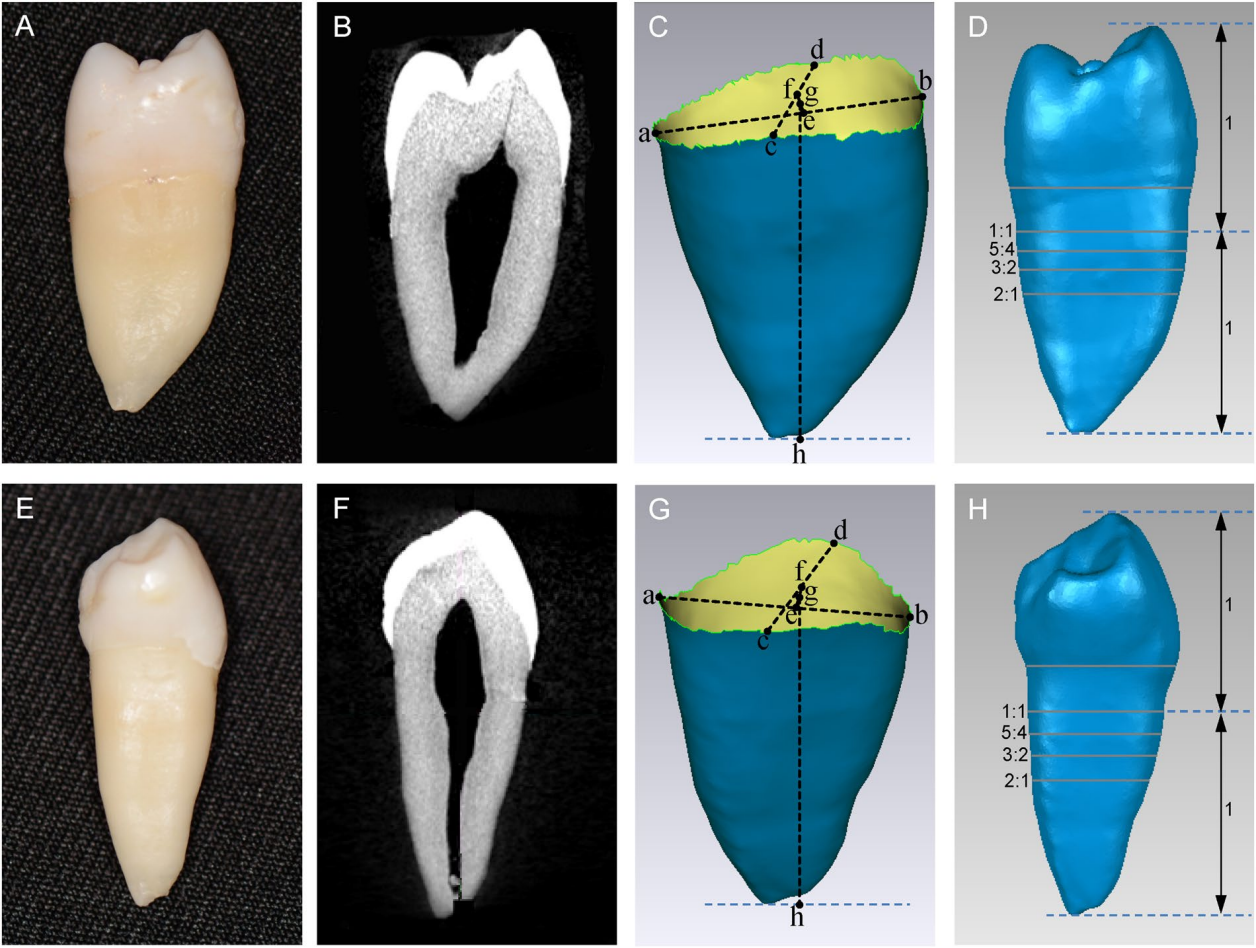
(**A**‒**D**): The views and estimated levels of a micro-CT scanned maxillary premolar. (**A**): maxillary premolar; (**B**): a sagittal view; (**C**): a–b: a line connecting the buccal and lingual CEJ, c–d: a line connecting the mesial and distal CEJ, e: the midpoint of a–b, f: the midpoint of c–d, g: the midpoint of e–f, representing the CEJ from a 2D perspective, and g–h: representing the 2D RL; (**D**): The CRRs were evaluated from 1:1 to 2:1. (**E**‒**H**): The views and evaluated levels of a micro-CT scanned mandibular premolar. (**E**): mandibular premolar; (**F**): the same as (**B**); (**G**): the same as (**C**); (**H**): The CRRs were evaluated from 1:1 to 2:1.

A total of 31 maxillary and 35 mandibular premolars were collected from patients undergoing orthodontic or periodontal treatment at the Dental Department of CGMH. The teeth were cleaned and scanned. The attained raw data of tooth structures were obtained as 2D images, which were assembled and converted into 3D reconstructions (Fig. 1). The 3D RSAs at the evaluated linear 2D CRRs were analyzed.

The results revealed that for the maxillary premolars, average 2D linear tooth length was 19.81 ± 0.31 mm (ranging from 16.4 mm to 23.0 mm), 100% RSA was 226.16 ± 5.99 mm^2^ (ranging from 173.1 mm^2^ to 299.5 mm^2^), total RL was 12.63 ± 0.19 mm (ranging from 10.8 mm to 14.4 mm), and 2D linear anatomical CRR was 1:1.83 ± 0.08 (ranging from 1:1.38 to 1:3.57). For the mandibular premolars, average tooth length was 20.53 ± 0.28 mm (ranging from 17.3 mm to 24.2 mm), 100% RSA was 202.43 ± 4.37 mm^2^ (ranging from 161.2 mm^2^ to 277.1 mm^2^), total RL was 13.28 ± 0.27 mm (ranging from 10.3 mm to 17.0 mm), and anatomical CRR was 1:1.88 ± 0.07 (ranging from 1:1.28 to 1:3.11). A wide distribution pattern was observed for these four examined factors for both premolars; however, significant variation between maxillary and mandibular premolars was observed only for 100% 3D RSA (Table 1, *p* = 0.002).

**Table 1:** The loss amount and percentages according to 3D RSA and 2D RL at the evaluated CRRs.

	Maxillary premolars	Mandibular premolars	Maxilla vs. Mandible
	Mean ± SE (n = 31)	Mean ± SE (n = 35)	*P* < 0.05
Total tooth length	19.81 ± 0.31 mm	20.53 ± 0.28 mm	*p* = 0.092
RSA at 100% PAL	226.16 ± 5.99 mm^2^	202.43 ± 4.37 mm^2^	*p* = 0.002**
Root length	12.63 ± 0.19 mm	13.28 ± 0.27 mm	*p* = 0.056
CRR with 100% PAL	1:1.83 ± 0.08	1:1.88 ± 0.07	*p* = 0.626
RSA & RL at CRR = 1:1			
Lost 3D RSA mm^2^ & %	57.24 ± 2.54 mm^2^	57.83 ± 3.16 mm^2^	*p* = 0.122
	25.62 ± 1.22%	28.35 ± 1.23%	
Lost 2D RL mm & %	2.72 ± 0.14 mm	3.02 ± 0.18 mm	*p* = 0.567
	21.41 ± 0.97%	22.22 ± 1.00%	
3D RSA% vs. 2D RL%	*p* < 0.001	*p* < 0.001	
RSA & RL at CRR = 5:4			
Lost 3D RSA mm^2^ & %	91.55 ± 3.51 mm^2^	86.72 ± 4.60 mm^2^	*p* = 0.516
	40.88 ± 1.65%	42.35 ± 1.53%	
Lost 2D RL mm & %	3.82 ± 0.14 mm	4.16 ± 0.18 mm	*p* = 0.567
	30.15 ± 0.86%	30.86 ± 0.89%	
3D RSA% vs. 2D RL%	*p* < 0.001	*p* < 0.001	
RSA & RL at CRR = 3:2			
Lost 3D RSA mm^2^ & %	109.22 ± 3.24 mm^2^	101.20 ± 4.06 mm^2^	*p* = 0.599
	48.67 ± 1.34%	49.67 ± 1.32%	
Lost 2D RL mm & %	4.71 ± 0.14 mm	5.07 ± 0.19 mm	*p* = 0.567
	37.13 ± 0.77%	37.77 ± 0.80%	
3D RSA% vs. 2D RL%	*p* < 0.001	*p* < 0.001	
RSA & RL at CRR = 2:1			
Lost 3D RSA mm & %	135.32 ± 3.66 mm^2^	124.51 ± 4.58 mm^2^	*p* = 0.532
	60.07 ± 1.06%	61.15 ± 1.31%	
Lost 2D RL mm & %	6.03 ± 0.14 mm	6.44 ± 0.20 mm	*p* = 0.567
	47.61 ± 0.64%	48.15 ± 0.67%	
3D RSA% vs. 2D RL%	*p* < 0.001	*p* < 0.001	

Independent t test for maxilla vs. mandible: *p < 0.05, **p < 0.01, ***p < 0.001.

Paired *t* tests for 2D RL % vs. 3D RSA %: *p < 0.05, **p < 0.01, ***p < 0.001.

Corresponding to linear 2D CRRs of 1:1, 5:4, 3:2, and 2:1, the results of the 3D RSA survey presented coronal bone support (in mm^2^) reductions of 25.62% ± 1.22%, 40.88% ± 1.65%, 48.67% ± 1.34%, and 60.07% ± 1.06%, respectively, for maxillary premolars and coronal bone support (in mm^2^) reductions of 28.35% ± 1.23%, 42.35% ± 1.53%, 49.67% ± 1.32%, and 61.15% ± 1.31%, respectively, for mandibular premolars (Table 1).

However, corresponding to 2D CRRs of 1:1, 5:4, 3:2, and 2:1, the 2D RL data presented coronal root length (in mm) reductions of 21.41% ± 0.97%, 30.15% ± 0.86%, 37.13% ± 0.77%, and 47.61% ± 0.64%, respectively, for maxillary premolars and coronal root length (in mm) reductions of 22.22% ± 1.0%, 30.86% ± 0.89%, 37.77% ± 0.80%, and 48.15% ± 0.67%, respectively, for mandibular premolars. The reduced percentages of 2D CRRs and 3D RSAs showed significant differences at all evaluated 2D CRR levels (Table 1, *p* < 0.001). Maxillary premolars and mandibular premolars demonstrated non-significant differences at the examined levels for both 3D RSAs and 2D CRRs (*p* ≥ 0.05). The variation between the measured and proposed amounts of bone support also revealed non-significant differences at the examined 2D CRR levels (Table 2, H0).

**Table 2:** The significance of the measured and proposed rates of 3D RSA and 2D RL at the evaluated CRRs.

	Maxillary premolars		Mandibular premolars	
	Measured rate Mean ± SE (n = 31)	Proposed rate (H0/H1)	Measured rate Mean ± SE (n = 35)	Proposed rate (H0/H1)
CRR = 1:1				
3D RSA%	25.62 ± 1.22%	vs. 25% (H0)	28.35 ± 1.23%	vs. 25% (H0)
2D RL%	21.41 ± 0.97%	vs. 25% (H0)	22.22 ± 1.00%	vs. 25% (H0)
CRR = 5:4				
3D RSA%	40.88 ± 1.65%	vs. 40% (H0)	42.35 ± 1.53%	vs. 40% (H0)
2D RL%	30.15 ± 0.86%	vs. 30% (H0)	30.86 ± 0.89%	vs. 30% (H0)
CRR = 3:2				
3D RSA%	48.67 ± 1.34%	vs. 50% (H0)	49.67 ± 1.32%	vs. 50% (H0)
2D RL%	37.13 ± 0.77%	vs. 40% (H0)	37.77 ± 0.80%	vs. 40% (H0)
CRR = 2:1				
3D RSA%	60.07 ± 1.06%	vs. 60% (H0)	61.15 ± 1.31%	vs. 60% (H0)
2D RL%	47.61 ± 0.64%	vs. 50% (H0)	48.15 ± 0.67%	vs. 50% (H0)

H0: no significance between the measured and proposed ratios of 3D RSA and 2D RL at evaluated CRRs.

In relation to 15%, 25%, 30%, 35%, 40%, 50%, and 60% coronal 2D RL bone losses, maxillary premolars displayed increased 2D CRRs of 1:1.21, 1:0.93, 1:0.82, 1:0.72, 1:0.63, 1:0.47, and 1:0.34, respectively. Correspondingly, when these coronal ratios of 2D RL bone losses were correlated with 3D RSA bone losses, the maxillary premolars also revealed increased 2D CRRs of 1:1.30, 1:1.05, 1:1.02, 1:0.92, 1:0.83, 1:0.63, and 1:0.52, respectively. Similarly, mandibular premolars revealed increased 2D CRR ratios of 1:1.24, 1:0.95, 1:0.83, 1:0.73, 1:0.64, 1:0.48, and 1:0.35, respectively, and increased 3D RSAs ratios of 1:1.35, 1:1.10, 1:1.04, 1:0.94, 1:0.85, 1:0.65, and 1:0.53, respectively, at the studied percentages. However, the 2D CRR ratios at the studied 2D RL levels were significantly different from the CRR ratios at the studied 3D RSA levels for both maxillary and mandibular premolars (Table 3, *p* < 0.05). Maxillary and mandibular premolars demonstrated a non-significant difference at the studied levels for both 3D RSA and 2D RL comparisons (*p* ≥ 0.05). The difference between the measured and proposed amounts of 2D CRRs also revealed a non-significant difference at the studied 2D RL and 3D RSA levels (Table 4, H0), except for the CRRs at 25% 2D RL for maxillary premolars and at 25% 3D RSA for mandibular premolars (Table 4, H1).

**Table 3:** The CRR at the evaluated 2D RL and 3D RSA levels.

Evaluated 3D & 2D levels	Maxillary premolars (n = 31)	Mandibular premolars (n = 35)	Maxilla vs. Mandible
	Mean ± SE	Mean ± SE	*P* < 0.05
CRR at 15% 3D RSA loss	1: 1.30 ± 0.04	1: 1.35 ± 0.04	*p* = 0.363
CRR at 15% 2D RL loss	1: 1.21 ± 0.04	1: 1.24 ± 0.04	*p* = 0.586
3D vs. 2D (*P* < 0.05)	*p* = 0.009	*p* < 0.001	
CRR at 25% 3D RSA loss	1: 1.05 ± 0.03	1: 1.10 ± 0.03	*p* = 0.197
CRR at 25% 2D RL loss	1: 0.93 ± 0.03	1: 0.95 ± 0.02	*p* = 0.574
3D vs. 2D (*P* < 0.05)	*p* < 0.001	*p* < 0.001	
CRR at 30% 3D RSA loss	1: 1.02 ± 0.04	1: 1.04 ± 0.03	*p* = 0.600
CRR at 30% 2D RL loss	1: 0.82 ± 0.02	1: 0.83 ± 0.02	*p* = 0.570
3D vs. 2D (*P* < 0.05)	*p* < 0.001	*p* < 0.001	
CRR at 35% 3D RSA loss	1: 0.92 ± 0.04	1: 0.94 ± 0.03	*p* = 0.708
CRR at 35% 2D RL loss	1: 0.72 ± 0.02	1: 0.73 ± 0.02	*p* = 0.566
3D vs. 2D (*P* < 0.05)	*p* < 0.001	*p* < 0.001	
CRR at 40% 3D RSA loss	1: 0.83 ± 0.03	1: 0.85 ± 0.03	*p* = 0.705
CRR at 40% 2D RL loss	1: 0.63 ± 0.01	1: 0.64 ± 0.01	*p* = 0.564
3D vs. 2D (*P* < 0.05)	*p* < 0.001	*p* < 0.001	
CRR at 50% 3D RSA loss	1: 0.63 ± 0.02	1: 0.65 ± 0.02	*p* = 0.271
CRR at 50% 2D RL loss	1: 0.47 ± 0.01	1: 0.48 ± 0.01	*p* = 0.560
3D vs. 2D (*P* < 0.05)	*p* < 0.001	*p* < 0.001	
CRR at 60% 3D RSA loss	1: 0.52 ± 0.02	1: 0.53 ± 0.02	*p* = 0.800
CRR at 60% 2D RL loss	1: 0.34 ± 0.01	1: 0.35 ± 0.01	*p* = 0.557
3D vs. 2D (*P* < 0.05)	*p* < 0.001	*p* < 0.001	

Independent t test for maxilla vs. mandible**:** *p < 0.05, **p < 0.01, ***p < 0.001.

Paired *t* tests for the CRRs of 3D RSA vs. 2D RL: *p < 0.05, **p < 0.01, ***p < 0.001.

**Table 4:** The significance of the measured and proposed rates of CRRs at the evaluated 3D RSA and 2D RL levels.

2D RL levels	Maxillary premolars		Mandibular premolars	
	Measured CRR Mean ± SE (n = 31)	Proposed rate (H0/H1)	Measured CRR Mean ± SE (n = 35)	Proposed rate (H0/H1)
at 15% 3D RSA	1: 1.30 ± 0.04	vs. 3:4 (H0)	1: 1.35 ± 0.04	vs. 3:4 (H0)
at 15% 2D RL	1: 1.21 ± 0.04	vs. 4:5 (H0)	1: 1.24 ± 0.04	vs. 4:5 (H0)
at 25% 3D RSA	1: 1.05 ± 0.03	vs. 1:1 (H0)	1: 1.10 ± 0.03	vs. 1:1 (H1)
at 25% 2D RL	1: 0.93 ± 0.03	vs. 1:1 (H1)	1: 0.95 ± 0.02	vs. 1:1 (H0)
at 30% 3D RSA	1: 1.02 ± 0.04	vs. 1:1 (H0)	1: 1.04 ± 0.03	vs. 1:1 (H0)
at 30% 2D RL	1: 0.82 ± 0.02	vs. 5:4 (H0)	1: 0.83 ± 0.02	vs. 5:4 (H0)
at 35% 3D RSA	1: 0.92 ± 0.04	vs. 1:1 (H0)	1: 0.94 ± 0.03	vs. 1:1 (H0)
at 35% 2D RL	1: 0.72 ± 0.02	vs. 4:3 (H0)	1: 0.73 ± 0.02	vs. 4:3 (H0)
at 40% 3D RSA	1: 0.83 ± 0.03	vs. 5:4 (H0)	1: 0.85 ± 0.03	vs. 5:4 (H0)
at 40% 2D RL	1: 0.63 ± 0.01	vs. 3:2 (H0)	1: 0.64 ± 0.01	vs. 3:2 (H0)
at 50% 3D RSA	1: 0.63 ± 0.02	vs. 3:2 (H0)	1: 0.65 ± 0.02	vs. 3:2 (H0)
at 50% 2D RL	1: 0.47 ± 0.01	vs. 2:1 (H0)	1: 0.48 ± 0.01	vs. 2:1 (H0)
at 60% 3D RSA	1: 0.52 ± 0.02	vs. 2:1 (H0)	1: 0.53 ± 0.02	vs. 2:1 (H0)
at 60% 2D RL	1: 0.34 ± 0.01	vs. 3:1 (H0)	1: 0.35 ± 0.01	vs. 3:1 (H0)

H0: No significance between the measured and proposed CRR at the evaluated 3D RSA and 2D RL levels.

H1: Significance between the measured and proposed CRR at the evaluated 3D RSA and 2D RL levels.

## Discussion

A wide distribution pattern was observed in tooth size and tooth length for the maxillary and mandibular premolars, and the mandibular premolars exhibited a significantly lower total RSA than the maxillary premolars. The present study applied percentages and ratios to calibrate individual teeth before analyzing the CRRs, and the mandibular and maxillary teeth were analyzed separately to avoid tooth size and anatomical biases.

Many factors, including initial patient assessment, periodontal disease severity, furcation involvement, etiological factors, restorative factors, and other determinants should be considered before retaining or extracting an abutment tooth in dental treatment^14^. The CRR clinical parameter, a restorative factor, is crucial for assigning a prognosis and predicting the survival rate of teeth experiencing alveolar bone support loss^2–5,14,19,20^. Clinical limitations of 2D radiographs including the superimposition of anatomical structures and burnout artifacts on radiographic film, as well as distorted images caused by the deviated angulation of the central radiation beam, could impede the operator’s ability to scan the teeth, reconstruct the images and analyze the recreated 3D RSA data. Moreover, few studies have discussed the association between CRR, periodontitis classification and the amount of periodontal attachment loss on the basis of 3D and 2D comparisons. The present study revealed a nonsignificant variation between the studied maxillary and mandibular premolars at the evaluated CRRs after calibrating the percentages.

### From a 2D RL Perspective

From a 2D radiographic perspective, when premolars experienced coronal 2D RL bone losses of 21.41%–22.22% (approximately 21.8%) at CRR = 1:1, the amount of bone loss was associated with the disease classification of moderate periodontitis^13^. However, when 1.0 mm of connective tissue attachment (CTA) was considered^21^, teeth with 2.72–3.02 mm of bone loss at CRR = 1:1 could be diagnosed as having mild periodontitis. Similarly, premolars with coronal 2D RL bone losses of 30.15%–30.86% at a CRR of 5:4 corresponded to the AAP disease classification of severe periodontitis (>30% or >5 mm radiographic bone loss or >5 mm CTA); the diagnosis became moderate periodontitis when 1.0 mm CTA was included in the periodontal support. Measured ratios of 37.13%–37.77% at a CRR of 3:2 demonstrated a nonsignificant variation when compared to the proposed 40% 2D ratio (H0, Table 2); therefore, when a tooth lost >5 mm coronal bone attachment at CRR ≥ 3:2 as shown on a 2D periapical film that included 1.0 mm CTA, a severe periodontitis diagnosis could be assigned. Consequently, depending on the inclusion or exclusion of 1.0 mm CTA, a periodontitis-affected tooth could be given two inconsistent diagnoses.

### From a 3D RSA Perspective

All 3D RSA and 2D RL percentages significantly differed at the evaluated CRRs (1:1, 5:4, 3:2, and 2:1) for both types of premolar (*p* < 0.001, Table 1), confirming that premolar roots that tapered from the cemento-enamel junction (CEJ) and coronal portion of the tooth root retain more 3D periodontal attachment. From a 3D RSA standpoint, a moderate periodontitis diagnosis could be assigned at a CRR value of 1:1, and a severe periodontitis diagnosis could be assigned at CRR values of 5:4, 3:2, and 2:1, with or without the inclusion of 1.0 mm CTA.

On the basis of the evaluated factors, such as if the percentages of radiographic bone loss included 1.0 mm CTA, assessing the periodontitis severity of a tooth was difficult because some CRR inconsistencies existed between the 2D RL and 3D RSA measurements. When the disease classification was evaluated according to CRR values and the percentages of radiographic bone loss (excluding 1.0 mm CTA), a case of mild periodontitis according to the AAP classification should correspond to 15% 2D RL bone loss at the proposed CRR level of 4:5. Inconsistently, 15% coronal 3D RSA bone loss occurred at the proposed CRR level of 3:4 (*p* = 0.009, Tables 3 and 4). Similarly, 30% 3D RSA support bone loss at a CRR of 1:1 and 25% 2D RL bone loss at a CRR of 5:4 represented a 30% radiographic bone loss and characterized moderate or severe periodontitis. Bone loss was highly significant at 30% 2D RL but not at 30% 3D RSA (*p* < 0.001, Table 2).

### CRRs and Periodontal Prognosis

Based on the classification of periodontal prognosis provided by McGuire^3,4^ in 1996, a fair prognosis can be assigned to a tooth that has lost approximately 25% clinical and radiographic periodontal attachment, and a poor or questionable prognosis can be assigned to teeth with >50% periodontal attachment loss. However, the role of 1.0 mm periodontal CTA should be considered when discussing CRRs and periodontal attachment. Bone loss of approximately 1.0 mm according to 2D RL was comparable with a value of approximately 10% according to 3D RSA CTA for the coronal half of the root (Table 1). A tooth needed to lose 25% periodontal attachment and 35% bone support at two CRRs to match the definition of 25% CAL. CRR values of 1:0.92–0.94 (35% 3D RSA loss) and 4:3 (35% 2D RL loss) indicated teeth with a fair periodontal prognosis according to the CAL perspective. When the assigned prognosis was based on 2D radiographic findings and 1.0 mm CTA was excluded, CRR values of 1:1.05–1.1 (25% 3D RSA loss) or CRR values of 1:0.93–0.95 (25% 2D RL loss) indicated teeth with a fair prognosis that lost 25% 3D RSA and 2D RL radiographic bone support, respectively. Similarly, CRR values of 2:1 (60% 3D RSA bone loss and 50% CAL) and CRR values of 3:1 (60% 2D RL bone loss and 50% CAL) indicated teeth with a poor or questionable prognosis. When 1.0 mm CTA was excluded, CRR values of 3:2 (50% 3D RSA loss) and CRR values of 2:1 (50% 2D RL loss) indicated teeth with a poor or questionable prognosis experiencing 50% 3D RSA and 2D RL radiographic bone support losses, respectively. A more coronal position of the measured 3D RSA level supported a favorable CRR expression in terms of periodontal prognosis. However, periodontal attachment losses were more severe when based on the 3D RSA measurement than when based on the 2D RL measurement at all evaluated CRRs. The tapering of the root anatomy corono-apically explains the results. Prosthodontists have concluded that the survival rate for prosthetic abutment teeth with CRRs of 5:4–3:2 are significantly lower according to a 7-year follow-up RPD study^22^. The levels at CRR values of 5:4–3:2 are comparable to the levels of severe periodontitis as defined by the AAP based on a 2D measurement. Therefore, when abutment teeth with severe periodontitis experienced >30%–40% 2D RL losses or 40%–50% 3D RSA radiographic bone losses at CRR values of >5:4–3:2, the prosthetic prognosis of the abutment teeth was decreased significantly. However, the preparation conditions of FPD, RPD abutment teeth, and nature teeth with periodontitis are different. In addition to the CRR, other parameters, including occlusal force, pier abutment teeth, and endodontic treatment, as well as other factors, such a significant variation in the 3D RSA and 2D RL periodontal attachment losses, should be considered before assigning a prognosis to teeth with compromised periodontal attachment. In addition, micro-CT analysis still mainly relies on laboratory studies, and the results of this study can only improve the diagnosis and understanding of conventional 2D radiographs. Clinically available 3D data using cone beam-CT analysis also have several limitations (e.g., scattering, noise, and movement artifacts). The risks associated with high radiation doses also restrict the clinical application of micro-CT in routine human physical examinations; conventional 2D radiographs will be continuously indicated for basic dental examination and diagnostics – including periodontal diseases. Other types of teeth, such as incisors, canines, and molars, may demonstrate various CRR characteristics and thus warrant additional studies.

## Conclusions

Based on 2D RL and AAP aspects, depending on whether radiographic bone loss is analyzed as a percentage or in terms of millimeters and on whether 1.0 mm CTA is included, a CRR of 1:1 corresponds to mild to moderate periodontitis, a CRR of 5:4 corresponds to moderate periodontitis, and CRRs of ≥3:2 correspond to severe periodontitis. However, when only the amount of radiographic bone loss is considered, CRRs of ≥5:4 are associated with severe periodontitis from a 3D perspective. In addition, a fair prognosis was found for premolars with 2D RL at a CRR of 4:3 and with 3D RSA at a CRR of 1:1 having 25% periodontal attachment and 35% bone support losses. Similarly, CRRs of 3:1 and 2:1 were associated with teeth with questionable prognoses with 50 and 60% periodontal attachments and bone support losses according to the 2D and 3D data, respectively. Taken together, these findings indicate that the systems that are currently applied to determine periodontitis severity and/or to assign periodontal-prosthetic prognoses for dentistry are asymmetrical according to the 2D and 3D viewpoints. Despite the limitations of the present study, we conclude that the 3D RSA and 2D RL measurements of single-rooted human premolars present inconsistent results in terms of the amount of bone support at the evaluated CRRs, regardless of whether CTA is included.

## Methods

Thirty-six extracted and intact human maxillary premolars and 35 mandibular single-rooted premolars were collected from patients with trauma or periodontitis or from patients who underwent orthodontic treatment at the Dental Department of Chang Gung Memorial Hospital (CGMH). This clinical study was conducted in conformity with the Declaration of Helsinki and was approved by the Medical Ethics Committee of Chang Gung Memorial Hospital. All methods were performed in accordance with the Taiwan Dental Association guidelines and regulations. All patients provided written informed consent.

Tooth samples were examined from the cuspid to the root apex using micro-CT (SkyScan 1076, Bruker, Kontich, Belgium) based on a cone beam method with the following settings: tube voltage, 100 kV; tube current, 100 µA; pixel matrix, 2,000 × 2,000; slice thickness, 18 μm. The apex of each premolar was stabilized vertically on a fixture that was arranged parallel to the long axis of the tooth. Generally, 20 minutes were required to scan each premolar. Subsequently, files were exported from the micro-CT instrument in digital imaging and communications in medicine and tagged image file data formats, in which a 5-GB file stored 1,300–1,500 slice images obtained for each tooth. These exported data were subsequently analyzed and edited using DataViewer, CTVol, and CTVox software packages (Bruker, Kontich, Belgium). 3D reconstructions were built from 2D images using the software. 3D geometry files were generated for each mask and were saved as stereolithography (STL) files. An STL format model including approximately 1,500,000–2,000,000 fine triangle surfaces describing a 3D premolar structure was developed. The RSAs of premolars were calculated as the sum of specific fine triangle areas using Pro/Engineer software (PTC, Needham, MA, USA).

The 2D RL of a scanned premolar was determined by measuring the distance from the apex to an average CEJ point (g–h in Fig. 1B and E); the CEJ point was defined as the midpoint of the two midpoints of the interproximal and buccolingual lines (f and e, respectively, in Fig. 1).

The loss amount and percentage of 3D RSAs at the evaluated CRRs (1:1, 5:4, 3:2, and 2:1) were analyzed. In addition, CRRs with 15%, 30%, and 50% coronal 2D RL bone loss and coronal 3D periodontal RSA bone loss were measured and evaluated. The collected teeth were assigned to maxillary and mandibular groups.

### Statistical Analyses

Both maxillary and mandibular premolars were analyzed after calculating the ratio of the RSA loss at planned levels to the total RSA loss amounts for individual teeth before performing intra- or inter-group analyses to avoid tooth size and root morphology bias.

Independent sample t-tests were conducted to evaluate the significance of differences in general anatomical measurement between the maxillary and mandibular premolars. Independent samples t-tests were also performed to study differences in the 2D RL and 3D RSA bone loss measurements between the maxillary and mandibular premolars at the corresponding CCRs (*p* < 0.05).

Paired t-tests were used to explore the significance of differences between the 2D RL and 3D RSA losses at the evaluated CRRs and 2D RLs, corono-apically (*p* < 0.05).

To simplify the comparison and discussion, a one-sample t-test was performed to assess whether the amount and ratio of bone attachment between the measured and proposed ratios at the evaluated CRRs, 3D RSAs, and 2D RLs supported the null hypothesis.

First H0: The variation between the measured and proposed amounts of bone support at CRRs of 1:1, 5:4, 3:2, and 2:1 based on the 3D RSA or 2D RL measurements were ≤2% (|mean−25% (or 30, 40, 50, and 60%)| ≤2%). First H1: the values were >2%.

Second H0: The variation between the measured and proposed ratios of bone support at linear 2D RLs or 3D RSAs of 15%, 25%, 30%, 35%, 40%, 50%, and 60% were ≤2% (|mean−15% (or 25, 30, 35, 40, 50, and 60%)| ≤2%). Second H1: the values were >2%.

## References

[1] Nunn ME (2012). Development of prognostic indicators using classification and regression trees for survival. Periodontol. 2000..

[2] McGuire MK (1991). Prognosis versus actual outcome: a long-term survey of 100 treated periodontal patients under maintenance care. J Periodontol..

[3] McGuire MK, Nunn ME (1996). Prognosis versus actual outcome. II. The effectiveness of clinical parameters in developing an accurate prognosis. J. Periodontol..

[4] McGuire MK, Nunn ME (1996). Prognosis versus actual outcome. III. The effectiveness of clinical parameters in accurately predicting tooth survival. J. Periodontol..

[5] Novak, K. F. & Takei, H. H. Determination of prognosis in *Carranza’s* Clinical *Periodontology* (ed. Newman, M. G., Takei, H., Klokkevold, P. R. & Carranza, F. A.) 373–383 (Elsevier, 2014).

[6] The Glossary of Prosthodontic Terms. *J Prosthet Dent*. **94**, 10–92 (2005). 10.1016/j.prosdent.2005.03.013 16080238

[7] Nyman SR, Lang NP (1994). Tooth mobility and the biological rationale for splinting teeth. Periodontol 2000..

[8] Grossmann Y, Sadan A (2005). The prosthodontic concept of crown-to-root ratio: a review of the literature. J. Prosthet. Dent..

[9] Schulte J, Flores AM, Weed M (2007). Crown-to-implant ratios of single tooth implant supported restorations. J. Prosthet. Dent..

[10] Dykema RW (1968). Fixed partial prosthodontics. J. Tenn. Dent. Assoc..

[11] Penny RE, Kraal JH (1979). Crown-to-root ratio: Its significance in restorative dentistry. J. Prosthet. Dent..

[12] Shillingburg, H. T., Hobo, S., Whitsett, L. D., Jacobi, R. & Brackett, S.E. Fundamentals of fixed prosthodontics. 85–103, 191–192 (Quintessence Publishing, 1997).

[13] Board AAP (2015). of Trustees. American Academy of Periodontology task force report on the update to the 1999 classification of periodontal diseases and conditions. J. Periodontol..

[14] Avila G (2009). A novel decision-making process for tooth retention or extraction. J. Periodontol..

[15] Van Staden RC, Guan H, Loo YC (2006). Application of the finite element method in dental implant research. Comput. Methods Biomech. Biomed. Engin..

[16] Kato A, Ohno N (2009). Construction of three-dimensional tooth model by micro-computed tomography and application for data sharing. Clin. Oral Invest..

[17] Smith TM (2009). Brief communication: dental development and enamel thickness in the Lakonis Neanderthal molar. Am. J. Phys. Anthropol..

[18] Parsa A, Ibrahim N, Hassan B, van der Stelt P, Wismeijer D (2015). Bone quality evaluation at dental implant site using multislice CT, micro-CT, and cone beam CT. Clin. Oral Implant Res..

[19] Ferraz C (2015). Effectiveness of different mechanical methods on dentin caries removal: micro-CT and digital image evaluation. Oper. Dent..

[20] McGuire MK, Nunn ME (1999). Prognosis versus actual outcome. IV. The effectiveness of clinical parameters and IL-1 Genotype in accurately predicting prognosis and tooth survival. J. Periodontol..

[21] Gargiulo AW, Wentz FM, Orban B (1961). Dimension and relation of dentogingival junction in humans. J. Periodontol..

[22] Tada S (2015). The impact of the crown-root ratio on survival of abutment teeth for denture. J. Dent. Res..

